# Lack of gene flow between *Phytophthora infestans* populations of two neighboring countries with the largest potato production

**DOI:** 10.1111/eva.12870

**Published:** 2019-09-28

**Authors:** Yan‐Ping Wang, Jia‐Hui Xie, E‐Jiao Wu, Lurwanu Yahuza, Guo‐Hua Duan, Lin‐Lin Shen, Hao Liu, Shi‐Hao Zhou, Oswald Nkurikiyimfura, Björn Andersson, Li‐Na Yang, Li‐Ping Shang, Wen Zhu, Jiasui Zhan

**Affiliations:** ^1^ State Key Laboratory of Ecological Pest Control for Fujian and Taiwan Crops Fujian Agriculture and Forestry University Fuzhou China; ^2^ Fujian Key Laboratory of Plant Virology Institute of Plant Virology Fujian Agriculture and Forestry University Fuzhou China; ^3^ Key Lab for Biopesticide and Chemical Biology Ministry of Education Fujian Agriculture and Forestry University Fuzhou China; ^4^ Department of Forest Mycology and Plant Pathology Swedish University of Agricultural Sciences Uppsala Sweden

**Keywords:** elongation factor‐1α (eEF‐1α), haplotype network, phylogenetic analysis, *Phytophthora infestans*, population connectivity, population genetic structure

## Abstract

Gene flow is an important evolutionary force that enables adaptive responses of plant pathogens in response to changes in the environment and plant disease management strategies. In this study, we made a direct inference concerning gene flow in the Irish famine pathogen *Phytophthora infestans* between two of its hosts (potato and tomato) as well as between China and India. This was done by comparing sequence characteristics of the eukaryotic translation elongation factor 1 alpha (eEF‐1α) gene, generated from 245 *P. infestans* isolates sampled from two countries and hosts. Consistent with previous results, we found that eEF‐1α gene was highly conserved and point mutation was the only mechanism generating any sequence variation. Higher genetic variation was found in the eEF‐1α sequences in the *P. infestans* populations sampled from tomato compared to those sampled from potato. We also found the *P. infestans* population from India displayed a higher genetic variation in the eEF‐1α sequences compared to China. No gene flow was detected between the pathogen populations from the two countries, which is possibly attributed to the geographic barrier caused by Himalaya Plateau and the minimum cross‐border trade of potato and tomato products. The implications of these results for a sustainable management of late blight diseases are discussed.

## 1 INTRODUCTION

Gene flow, referred to the movement of gametes, genotypes, or extranuclear segments of DNA such as mitochondria from one population to another through migration and hybridization event (Toews, Mandic, Richards, & Irwin, 2013), plays a dual role in the evolution of organisms (Slatkin, 1987). Evolutionary theory considers isolation as one of the essential steps leading to speciation. Regular gene flow acts as a constraining force on evolution by homogenizing genetic and phenotypic variation among populations (Booth Jones et al., 2017). On the other hand, occasional gene flow accelerates evolutionary processes by spreading successful genes or genotypes to neighboring populations (Paun, Schönswetter, Winkler, Tribsch, & Intrabiodiv, 2008). Under the shifting balance theory (Wright, 1990), gene flow is an essential factor in enforcing population replacements. In nature, biology (e.g., dispersal mechanism and reproductive mode), community composition (e.g., kinship and biotypes), and landscape structure (e.g., patch sizes and shapes) are among the most important elements influencing gene flow of organisms (Ropars et al., 2018). Habitat heterogeneity, genetic incompatibilities, and limited dispersal ability usually reduce gene flow among populations (Dong et al., 2016; Semizer‐Cuming, Kjaer, & Finkeldey, 2017). Human activities also regulate gene flow by influencing habitat connectivity, niche size, and long‐distance movement of organisms (Hu, Gao, & Zhu, 2017).

Knowledge of gene flow is important to understand the evolutionary history of species, as well as to predict their adaptive responses to future ecological and environmental fluctuations such as climate change (Edelaar & Bolnick, 2012; Garant, Forde, & Hendry, 2007). In the field of plant pathology, knowledge of gene flow of pathogens is critical for developing preventive and eradicative strategies to mitigate the epidemiological and evolutionary risks of infectious diseases in major crops (McDonald & Linde, 2002). The extent of gene flow is usually estimated indirectly based on the variance of allele frequencies among populations using *F*‐statistics (O'Donald, 1972). The indirect methods infer the average number of individuals that are successfully incorporated into the breeding system of resident populations (Korman et al., 1993; Panyamang, Duangphakdee, & Rattanawannee, 2018) and emphasize the long‐term effects of gene flow on semi‐isolated populations (Singh & Singh, 2008). However, these estimates are constrained by a large number of assumptions that are unlikely to be met in practice (Meirmans & Hedrick, 2011). Alternatively, gene flow can be inferred directly by detecting identical genotypes using DNA‐based molecular marker technologies. In practice, many molecular marker technologies can rapidly differentiate almost all genetically distinct genotypes in a population, even if a small number of marker loci is used to assay a large number of individuals (Cornuet, Piry, Luikart, Estoup, & Solignac, 1999). Furthermore, direct measurement of gene flow by genotype identification can provide important insight into the role of gene flow on the evolution of organisms over contemporary time scales (Rannala & Mountain, 1997).

However, the accuracy of direct estimations of gene flow by the detection of identical genotypes with molecular technologies is affected by marker resolution and mutation rate. Low‐resolution markers based on fragment sizes tend to overestimate the extent of gene flow because size homoplasy can misassign nonhomologous individuals in different populations to a same genotype (Caballero, Quesada, & Rolan‐Alvarez, 2008). High mutation rate in molecular markers can, on the one hand, facilitate the generation of analogous sequence structures from different ancestry genotypes, leading to an overestimate of gene flow among populations. This sequence convergence has been widely documented in all species kingdoms (Balloux, Lugon‐Moulin, & Hausser, 2000). On the other hand, it can also lead to an underestimation of the gene flow, caused by enhanced divergent evolution of identical genotypes in different populations. DNA sequencing technology provides the highest resolution of genotyping species (Lexer et al., 2016). As a consequence, detecting identical genotypes among populations by sequencing genes with a low evolutionary rate should provide more accurate direct estimates of gene flow.

As a housekeeping gene, elongation factor‐1α (eEF‐1α) is one of the most abundant and conserved sequences in eukaryotes (Hovemann, Richter, Walldorf, & Cziepluch, 1988). It encodes an isoform of the alpha subunit of the elongation factor‐1 complex, an essential component of the protein synthesis process. During protein synthesis, the factor‐1 complex forms a ternary structure with GTP and aminoacyl‐tRNA and delivers appropriate amino acids to the ribosome (Moldave, 1985). In addition to protein synthesis, the eEF‐1α subtype may be involved in functions such as organization of the mitotic apparatus, developmental regulation, signal transduction, aging, transformation, and immunoreactivity (Piedra‐Quintero, Apodaca‐Medina, Beltran‐Lopez, Leon‐Sicairos, & Lopez‐Moreno, 2015; Riis, Rattan, Clark, & Merrick, 1990). In the life cycle of species, eEF‐1α can be found in all developmental stages both in the cytoplasm and nucleus of cells (van't Klooster, 2000). Due to its universal occurrence, sufficient information, and slow rate of sequence evolution (Baldauf & Doolittle, 1997), the eEF‐1α gene and its translated product are well suited for determining phylogenetic relationships among species and quantifying host–pathogen interaction (Chen & Halterman, 2011). It can also be used to directly estimate gene flow among populations by detecting identical genotypes of a species.

*Phytophthora infestans* (Mont.) de Bary, the cause of late blight in potato and tomato, can affect all parts of crops in the field and storage (Haas et al., 2009). Fast epidemics and rapid evolution are among the main challenges to effectively and sustainably control this disease. If uncontrolled, late blight can destroy entire crops within just a few days under favorable climatic conditions (Fry et al., 2015). Although many management strategies have been developed and deployed to control it over the last decades, *P. infestans* is still among the most destructive plant pathogens, causing approximately 8 billion US dollars annually of economic losses worldwide in potato production alone (Runno‐Paurson et al., 2013). The pathogen is spread by rain‐splash, infected plant materials, and wind‐born sporangia (Fernández‐Pavía, Grünwald, Díaz‐Valasis, Cadena‐Hinojosa, & Fry, 2004; Judelson et al., 2008), and increasing global trade in potato products facilitates long‐distance spread and gene flow of the pathogen. This provides recurrent opportunities for new invasions of the pathogen (Montarry et al., 2010), enhancing its capacity of adaptation to changing environments and aggravating the difficulty to control it (Zhan, Thrall, & Burdon, 2014; Zhan, Thrall, Papaix, Xie, Burdon, 2015). Gene flow in a continental scale has been documented many times in *P. infestans* (Goodwin, Sujkowski, Dyer, Fry, & Fry, 1995). For example, the Blue‐13 lineage found first time in the Netherlands in 2004 (Cooke et al., 2012) is believed to have been rapidly spread to many others countries including China and India (Chowdappa et al., 2014). However, the knowledge of gene flow in *P. infestans* is primarily derived indirectly from population analysis of the pathogen using fragment technologies such as isozyme and RFLP. In order to verify these indirect inferences derived, direct detection of gene flow by identifying genotypes shared among populations by DNA sequencing of conserved genes is important.

In this study, we compared sequence characteristics of eEF‐1α gene generated from 245 *P. infestans* isolates originating from potato and tomato across wide geographic regions in China and India, the two largest potato production countries in the world. In 2017, the two countries produced 148 million tons of potato on around 8,000,000 hectares (http://www.fao.org/), accounting for 38% of total global potato production and 41% of the global potato acreage. Potato production in the two countries is still expanding.

The objectives of this study were to: (a) investigate population genetic structure of eEL‐1α gene in the late blight pathogen *P. infestans*; (b) determine the types of sequence variation in eEL‐1α gene; (c) infer the effect of host on the population genetic structure of *P. infestans*;and (d) infer gene flow of *P. infestans* between two countries with the largest potato production in the world and its implications for the sustainable management of the late blight disease.

## 2 MATERIALS AND METHODS

### 2.1 *Phytophthora infestans* eEF‐1α sequences

**Figure 1:**
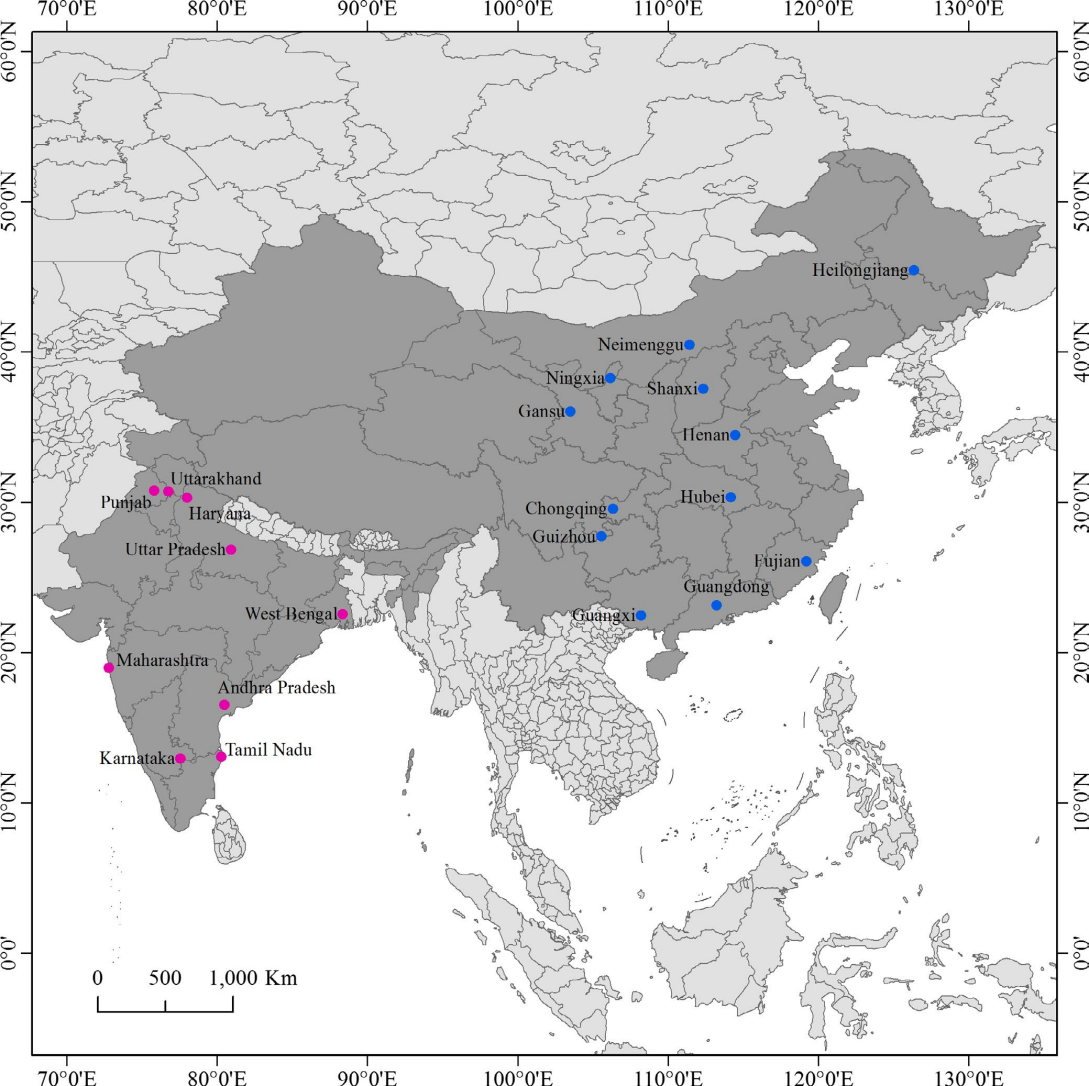
Map showing the geographic locations (blue) of the *Phytophthora infestans* populations included in the current study. ArcGIS 10.0 software was used to create the map. *Phytophthora infestans* isolates from China and India are indicated by blue and pink

A total of 245 eEF‐1α sequences were included in the current analysis of population genetic structure in *P. infestans*. Of these, 165 sequences were generated from 156 potato isolates and nine tomato isolates across China (Figure 1). The remaining 80 sequences, represented 48 potato isolates and 32 tomato isolates from India (Figure 1), were retrieved from GenBank (Table S1, Nirmal Kumar, Chowdappa, & Krishna, 2016). The isolates from China were pregenotyped with molecular amplification of eight SSR markers (Knapova & Gisi, 2002; Lees et al., 2006), restriction enzyme‐PCR amplification of mitochondrial haplotypes (Flier et al., 2003), mating type (Zhu et al., 2015), and partial sequence analysis of three genes (b‐tubulin, Cox1 and Avr3a) (Cardenas et al., 2011). Only isolates with a distinct genotype were selected for sequencing. In both hosts, leaves with a typical late blight symptom were collected from plants separated by at least one meter and transported to the laboratory within 24 hr for pathogen isolation. Detailed information on the pathogen isolation can be found in previous publications (Yang et al., 2016; Zhu et al., 2016, 2015). Briefly, infected leaves were first rinsed with running water for 60 s and then with sterilized distilled water for 30 s. A piece of diseased tissues was cut from the margin of leaf lesions and placed abaxial side up on 2.0% water agar for 20–30 hr. A single piece of mycelium was removed aseptically from the sporulating lesions using an inoculating needle, transferred to a rye B agar plate supplemented with ampicillin (100 μg/ml) and rifampin (10 μg/ml), and maintained in the dark at 19°C for 7 days to allow a colony to develop. The isolates were purified by two sequential transfers of a single piece of mycelium hyphae tipped from the colony to a fresh rye B plate and maintained at 13°C until use.

To extract DNA, *P. infestans* isolates retrieved from a long‐term storage were cultured on rye B agar supplemented with ampicillin (100 μg/ml) and rifampin (10 μg/ml) at 19°C in the dark for 15 days. Mycelia were harvested, transferred into a sterile, 2‐ml centrifuge tube, and lyophilized with a vacuum freeze dryer (Alpha1‐2, Christ). The lyophilized mycelia were ground to powder with a mixer mill (MM400, Retsch), and genomic DNA was extracted using a plant genomic DNA kit (Promega Biotech. Co. TRANSGEN) according to the manufacturer's instructions. Approximately 100 mg mycelia were used for DNA extraction of each isolate. The extracted DNA was suspended in ultrapure water and kept in −40°C refrigerator until use.

The eEF1‐α gene in the genomic DNA from the *P. infestans* isolates was amplified by a pair of primers (F: 5′‐GCCATATACAGCTGAGAAATCTCA‐3′ and R: (5′‐CTGTACAGTAGATGAGAATCAGATG‐3′). PCR amplifications were performed in a total reaction volume of 50 μl composed of 1.0 μl HifiTaq DNA polymerase, 5.0 μl 10 × HiFi Buffer II, 4.0 μl of dNTPs (10 μmol/L), 2.0 μl of forward primer (10 μmol/L), 2.0 μl of reverse primer (10 μmol/L), 34 μl of ddH_2_O, and 2.0 μl of template DNA using a Gene CyclerTM (Bio‐Rad). The PCR program was started with an initial denaturation step of 95°C for 5 min; followed by 35 cycles of amplification for 30 s at 94°C, annealing at 61°C for 30 s, extension at 72°C for 60 s; and ended with a final extension step at 72°C for 5 min. The PCR products were separated on 1% agarose gels by electrophoresis, purified for single direction sequencing according to manufacturer's instructions (QIAquick® Gel Extraction Kit), ligated into a T1 zero cloning vector, and transformed into Trans1‐T1 competent cells by heat‐shock at 42°C for 30 s (pEASY®‐T1 Zero Cloning Kit). Colonies with single and expected band size were sent to GenScript Biological Technology Co., Ltd. (GenScript) for sequencing using an ABI3730 automated DNA sequencer (Applied Biosystems).

### 2.2 Data analysis

Nucleotide sequences were visually assessed to remove potential mutations caused by PCR artifacts (Suzan et al., 2007). Amino acid haplotypes were deduced from nucleotide sequences. The multiple sequence alignment of eEF‐1α gene was performed using the ClustalW algorithm embedded in MEGA 7.0.21 (Kumar, Stecher, & Tamura, 2016), and the mutation site map was generated by BioEdit Sequence Alignment Editor (Hall, 1999). Nucleotide haplotypes were constructed with the PHASRE algorithm implemented in DnaSP 5.10 (Librado & Rozas, 2009) and coded with the letter “H” followed by a corresponding number. Genetic variation in *P. infestans* populations was evaluated by nucleotide diversity, nucleotide haplotype diversity, and nucleotide haplotype richness and was estimated for each population as well as the combined population by pooling the nucleotide sequences for individual host and country using DNA Sequence Polymorphism Version 6.11.01. A median joining (MJ) haplotype network was generated by Dansp6 for nucleotide sequences and visualized by Popart v.1.7. Each nucleotide haplotype was represented by a circle, and the proportions of isolates with a particular nucleotide haplotype were indicated by circle sizes. Steps of nucleotide substitution between nucleotide haplotypes were indicated by number of tick marks.

Phylogenetic trees were reconstructed from unique eEF1‐α nucleotide haplotypes as well as all eEF1‐α sequences using the neighbor‐joining (NJ) method (Saitou & Nei, 1987) embedded in MEGA 7.0.21. The robustness of phylogenetic trees was evaluated by bootstrap test with 1,000 replicates. The evolutionary distance among phylogenetic branches was computed using the Maximum Composite Likelihood method and presented as the number of base substitutions per site.

## 3 RESULTS

### 3.1 Sequence variation in the eEF‐1α gene of *Phytophthora infestans*

**Figure 2:**
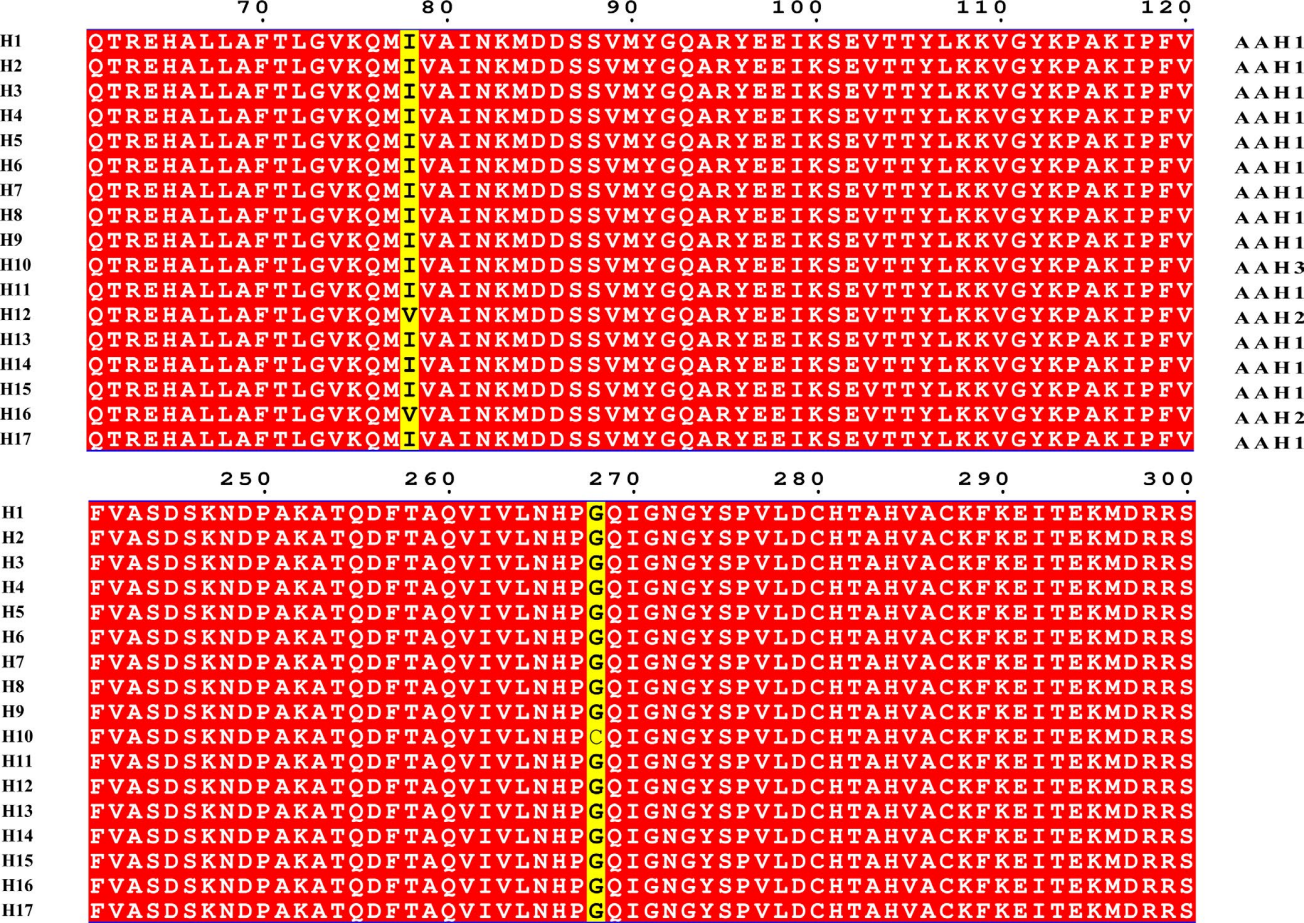
Sequence alignments of three amino acid (AA 61–120 and AA 241–300) haplotypes of the eEF‐1α gene deduced from 17 nucleotide haplotypes. Different amino acids in the partial sequences were shown in yellow and shared amino acids were shown in red. The sequence alignment was performed by a ClustalW multiple approach and displayed by the program ESPript

A total of 245 partial eEF‐1α sequences from China and India were included in the analysis of population genetic structure in *P. infestans*. Multiple sequence alignment indicates that all sequence variations were generated by point mutations (Figure 2). No introns were found in the eEF‐1α sequences. Since no deletions, duplications, or early‐terminations exist in the eEF‐1α gene, all sequences from different isolates are identical in size and the average nucleotide identities in the eEF‐1α sequences among all the studied *P. infestans* isolates were 99.9%.

Seventeen nucleotide haplotypes (Figure 2) generated by 18 mutation points (Table 1) were recovered from the 245 eEF‐1α sequences with an overall haplotype diversity of 0.62 and nucleotide diversity of 0.0049 when the nucleotide sequences from different countries and hosts were considered together (Table 2). Among the 18 mutation points, five are transversion and 13 are a transition (Table 1). No identical nucleotide haplotypes were detected in the *P. infestans* populations from the two countries, but haplotypes were shared among the *P. infestans* populations originated from different hosts within the same countries. The most common haplotype (H1) was detected in China, accounting for 58.78% of the combined population, while the second most common haplotype (H6) was recovered from India, accounting for 16.73% of the combined population (Table 3). When the nucleotide sequences were considered according to individual country, higher genetic variation was found in the eEF‐1α gene from India than China. A higher variation in the eEF‐1α gene was also found in samples from tomato compared to samples from potato when the samples from the two countries were combined (Table 2). In India, 12 nucleotide haplotypes were recovered from 80 sequences with an overall haplotype diversity of 0.70 and nucleotide diversity of 0.0016, while only five nucleotide haplotypes with a haplotype diversity of 0.23 and nucleotide diversity of 0.0004 were detected in the 165 sequences from China (Table 1). In tomato, 12 nucleotide haplotypes were recovered from 41 sequences with an overall haplotype diversity of 0.81 and nucleotide diversity of 0.0043, while only 11 nucleotide haplotypes with a haplotype diversity of 0.49 and nucleotide diversity of 0.0041 were detected in the 204 sequences from potato (Table 2).

**Table 1:** The positions and types of nucleotide substitution in the eEF‐1α haplotypes (H1‐17) of *Phytophthora infestans* sampled from potato and tomato in China and India

Positions and types of substitution	H1	H2	H3	H4	H5	H6	H7	H8	H9	H10	H11	H12	H13	H14	H15	H16	H17
87s	A	A	G	A	G	G	G	G	G	G	G	G	G	A	G	G	G
232s	A	A	A	A	A	A	A	A	A	A	A	G	A	A	A	G	A
243s	T	T	T	T	T	C	C	C	C	C	C	C	C	C	C	C	T
363s	C	C	C	C	C	T	T	T	T	T	T	T	T	C	T	T	T
396s	C	C	C	T	C	C	C	C	C	C	C	C	C	C	C	C	C
399s	C	C	T	C	T	C	C	C	C	C	C	C	C	C	C	C	C
414s	A	G	G	A	A	G	G	G	G	G	G	G	G	G	G	G	G
477v	T	T	T	T	T	G	G	G	T	T	T	G	G	T	T	G	T
507s	T	T	T	T	T	C	C	C	C	C	C	C	C	C	C	C	C
624s	C	C	C	C	C	T	T	T	T	T	T	T	T	C	C	C	T
654s	G	G	G	G	G	G	A	A	G	G	A	G	A	G	G	G	G
738v	G	G	G	G	G	C	C	C	C	C	C	C	C	C	C	C	C
802v	G	G	G	G	G	G	G	G	G	T	G	G	G	G	G	G	G
804s	C	C	C	C	C	T	T	T	T	T	T	T	T	T	T	T	T
825v	G	G	G	G	G	G	G	G	G	C	G	G	G	G	G	G	G
867s	C	C	C	C	C	T	C	C	T	T	T	T	T	T	T	T	T
918v	T	T	T	T	T	T	G	T	T	T	T	T	T	T	T	T	T
948s	C	C	C	C	C	C	T	C	C	C	C	C	C	C	C	C	C

Note

s = transition and v = transversion.

**Table 2:** Sample sizes and genetic variation in the eEF‐1α sequences of *Phytophthora infestans* populations sampled from potato and tomato in China and India

Populations	Sample size	Haplotype number	Haplotype diversity	Nucleotide diversity
China	165	5	0.23B	0.0004B
India	80	12	0.70A	0.0016A
Potato	204	11	0.49B	0.0041B
Tomato	41	12	0.81A	0.0043A
Total	245	17	0.62	0.0049

Note

Values in the same columns followed by different letters are significantly different at *p* = .01 level.

**Table 3:** Frequency distribution of nucleotide haplotype in the eEF‐1α sequences of *Phytophthora infestans* populations sampled from potato and tomato in China and India

Haplotypes	Country	Frequency		
		Potato	Tomato	Combined
H1	China	0.70	0.02	0.59
H2	China	0.04	0.05	0.04
H3	China	0.01	0.05	0.02
H4	China	0.00	0.10	0.02
H5	China	0.01	0.00	0.01
H6	India	0.14	0.32	0.17
H7	India	0.02	0.00	0.02
H8	India	0.02	0.00	0.01
H9	India	0.02	0.29	0.06
H10	India	0.02	0.00	0.01
H11	India	0.02	0.00	0.01
H12	India	0.01	0.02	0.01
H13	India	0.00	0.05	0.01
H14	India	0.00	0.02	0.00
H15	India	0.00	0.02	0.00
H16	India	0.00	0.02	0.00
H17	India	0.00	0.02	0.00
Total		1.00	1.00	1.00

Note

Sample size for each host and country is shown in Table 2.

Only three amino acid haplotypes (isoforms) were deduced from the 17 nucleotide haplotypes (Figure 2). The main amino acid haplotype (AAH1) deduced from nucleotide haplotypes H1–H9, H11, H13–15, and H17 was found in both *P. infestans* populations from China and India (Table 4). It was the only amino acid haplotype detected in China (100%) and also accounted for 90.12% of Indian *P. infestans* population. AAH2 deduced from H12 and H16 was generated by the substitution of isoleucine in the 78th amino acid of AAH1 with valine, while AAH 3, deduced from H10, was generated by the substitution of glycine in the 268th amino acid of AAH1 with cysteine. AAH2 and AAH3 were only found in the *P. infestans* population from India.

**Table 4:** Frequency distribution and deduced amino acid haplotypes of eEF‐1α in the *Phytophthora infestans* populations originated from China and India

Amino acid haplotypes	Deduced from	Frequency		
		China	India	Combined
AAH1	H1–H9, H11, H13–H15, H17	1.00	0.90	0.97
AAH2	H12, H16	0.00	0.05	0.02
AAH3	H10	0.00	0.04	0.01
Total		1.00	1.00	1.00

Note

Sample size for each host and country is shown in Table 2.

### 3.2 Haplotype network of eEF‐1α

**Figure 3:**
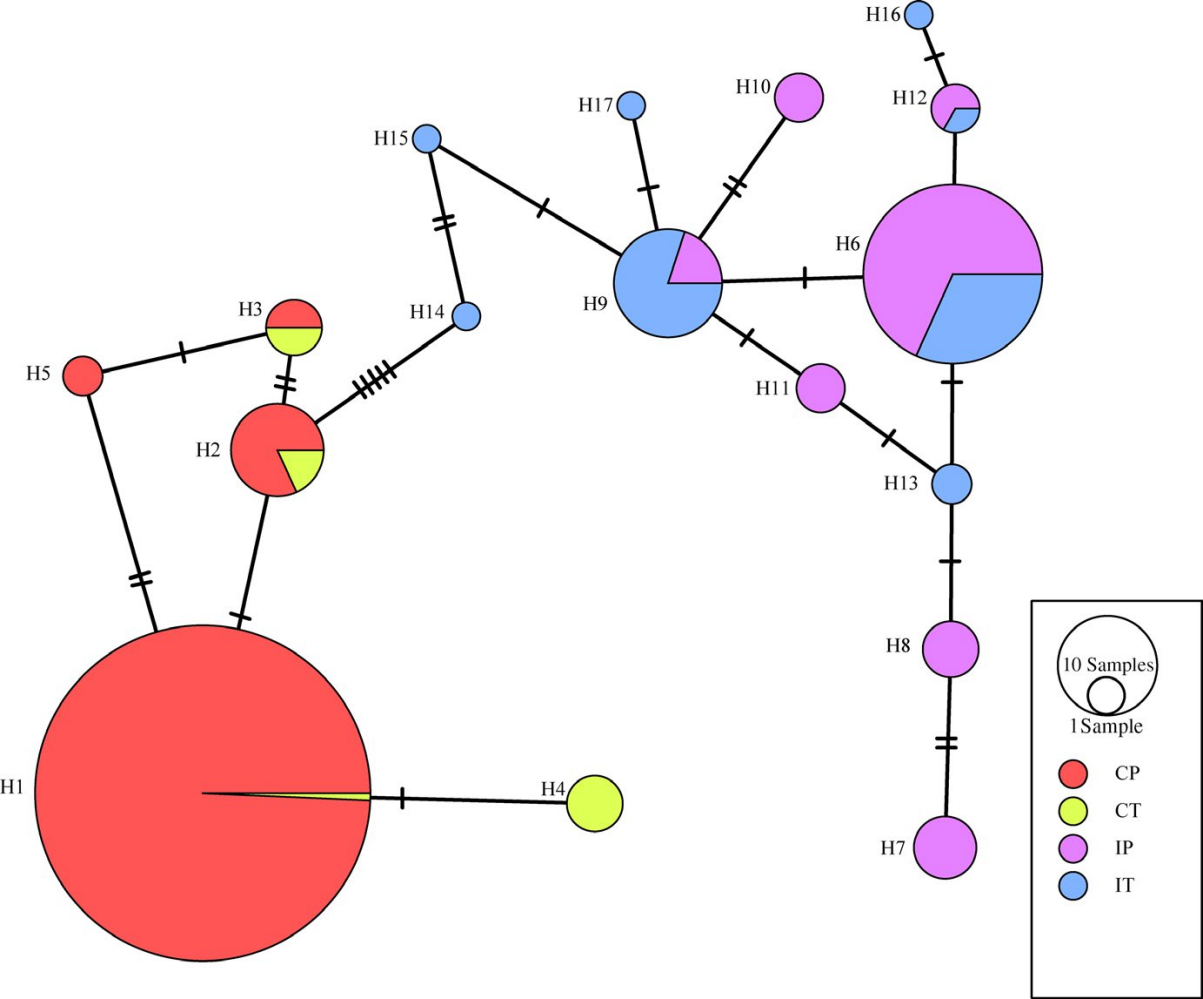
Nucleotide haplotype network of eEF‐1α gene in the *Phytophthora infestans* populations sampled from potato and tomato in China and India. The network was constructed by a maximum parsimony approach. Nucleotide haplotypes are named by the letter H followed by a corresponding number. Each circle represents a unique haplotype, and size of circles indicates the frequency of isolates with that particular haplotype. Each tick mark represents a step of nucleotide substitution. Black circles represent missing haplotypes

The nucleotide haplotype network of eEF‐1α gene formed two major groups and there was a clear geographic association among haplotypes (Figure 3). The less diverse group comprised of all five nucleotide haplotypes from China diverged by a maximum of three mutation steps among haplotypes. The other group was more diverse. It included all 12 nucleotide haplotypes from India and diverged by a maximum of eight mutation steps among haplotypes. The two groups were connected by five mutation steps. H1, the most abundant nucleotide haplotype in China, and H6, the most abundant nucleotide haplotype in India, were distanced by 10 mutation steps. All three nucleotide haplotypes (H10, H12, and H16) coding the rare amino acid haplotypes (AAH2 and AAH3) were located in the tip of the network tree. The nucleotide haplotype network also contained two reticulating structures. One reticulation was formed by four haplotypes (H1, H2, H3, and H5) from China, and another one was formed by other four haplotypes (H6, H9, H11, and H13) from India. Most nucleotide haplotypes were unevenly distributed between potato and tomato hosts.

### 3.3 Phylogenetic analysis

**Figure 4:**
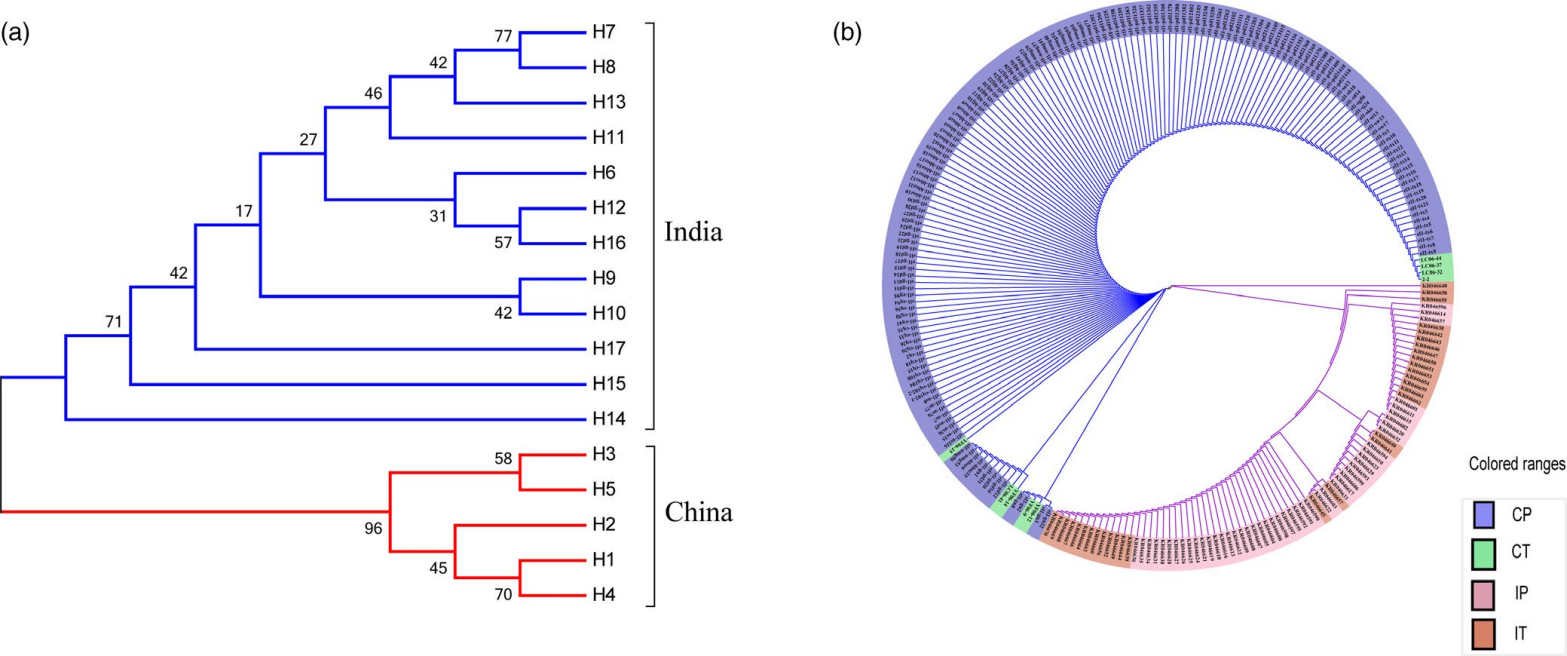
The phylogenetic trees of eEF‐1α sequences reconstructed by a neighbor‐joining (NJ) approach embedded in the MEGA 7.0.21 program. The bootstrap values were calculated from 1,000 replications: (a) The NJ tree based on 17 nucleotide haplotypes of eEF‐1α gene; and (b) the NJ tree based on all eEF‐1α nucleotide sequences from potato and tomato in China and India. CP, CT, IP, and IT represent eEF‐1α sequences from Chinese potato, Chinese tomato, Indian potato, and Indian tomato, respectively

Similar to network analysis, phylogenetic cluster analysis by a neighbor‐joining approach also produced a dendrogram that divided the 17 nucleotide haplotypes into two main clades with a significantly statistical support (Figure 4a). One of the two clades was composed of all nucleotide haplotypes from China, and the other was composed of all nucleotide haplotypes from India. Within the countries, nucleotide sequences from different hosts were clustered together into a monophyletic group (Figure 4b). There were fewer and shorter branches in the eEF‐1α sequences of *P. infestans* population from China than India, but there was no difference in the number and length of branches between the *P. infestans* sequences from potato or tomato (Figure 4).

## 4 DISCUSSION

Overall, a low genetic variation was found in the eEF‐1α gene. Only 17 nucleotide and three amino acid haplotypes were detected in the 245 sequences. The genetic variation is substantially lower than in many functional genes in *P. infestans* (Yang et al., 2018) and other pathogens (Marisa et al., 2018). For example, from a subset of the same Chinese collection, 51 nucleotide haplotypes were identified in the 96 Avr3a sequences (Yang et al., 2018). The eEF‐1α is a housekeeping gene playing multifaceted roles in the biochemical and physiological processes of life (Chang et al., 2002; Kato, Sato, Nagayoshi, & Ikawa, 1997). Low genetic variation in eEF‐1α is consistent with evolutionary hypothesis that genes important to cell functions evolve at a reduced rate (Jordan, Rogozin, Wolf, & Koonin, 2002).

Housekeeping genes routinely experience purified selection through which genetic variation is largely reduced (Viscidi & Demma, 2003). In addition, a lack of intragenic recombination may also contribute to the low genetic variation in the eEF‐1α gene. Intragenic recombination can generate new haplotype variation (Watt, 1972) and has been commonly found in genes responsible for the interaction of pathogens with hosts and other environments (Stergiopoulos et al.., 2013) including effector and fungicide resistance genes of *P. infestans* (Chen, Zhou, Qin, Li, & Zhan, 2018; Yang et al., 2018). Although some reticulation structures exist in haplotype network (Figure 3), no signals of intragenic recombination were identified in the eEF‐1α gene by any of the seven algorithms implemented in the RDP4 suite (data not shown). Thus, it is reasonable to believe that the reticulation structures were generated by convergent evolution of nucleotide sequences (Ralph & Coop, 2015), suggesting that mutations occur frequently in the eEF‐1α gene and the observed low genetic variation was likely caused by other mechanisms such as purifying selection rather than low mutation rate of the gene.

Higher genetic variation was found in the eEF‐1α sequences of *P. infestans* populations originating from India compared to populations from China. This result is consistent with previous surveys using a similar set of neutral markers. With the SSR markers, 24 multi‐locus genotypes were detected among 59 *P. infestans* isolates sampled from India (Dey et al., 2018), while only 26 multi‐locus genotypes were identified among 279 isolates sampled from China (Tian, Yin, Sun, Ma, Ma, Quan, et al., 2015; Tian, Yin, Sun, Ma, Ma, Wang, et al., 2015). China produces potato and tomato on larger acreages than India, and therefore, it is expected to host a larger *P. infestans* population than India. Pathogens with a larger population size tend to have a higher genetic variation due to more alleles being generated by mutations and fewer alleles being lost by genetic drift (Lázaro‐Nogal, Matesanz, García‐Fernández, Traveset, & Valladares, 2017). Furthermore, unlike the Indian material, isolates from China were prescreened molecularly and phenotypically and only isolates with distinct genotypes were selected for sequencing in this study. The fact that a higher genetic variation was still found in the Indian *P. infestans* population is likely caused by other natural factors and agricultural practices promoting the accumulation of genetic variation in the pathogen populations such as conducive environmental conditions, diversifying selection for different ecosystems, increasing international trade of plant materials, and reduced field hygiene during production (Dey et al., 2018). Indeed, it is reported that farmers in many parts of India tend to use potato tubers saved from previous years as seeds (Chowdappa et al., 2014; Dey et al., 2018) and new pathotypes constantly emigrate naturally from neighboring countries like Bangladesh and Nepal or even artificially from Europe (Dey et al., 2018). On the other hand, migration of *P. infestans* to China is limited (Tian, Yin, Sun, Ma, Ma, Wang, et al., 2015).

The *P. infestans* populations sampled from tomato displayed a higher genetic variation than those sampled from potato (Table 2), and this pattern of genetic difference among host origins was found in both China and India (data not shown). This is consistent with a previous result from France (Wangsomboondee, Groves, Shoemaker, Cubeta, & Ristaino, 2002) in which the higher genetic variation of *P. infestans* from tomato than potato was thought to be resulted from the different mating systems adopted by the pathogen on the two hosts, that is, sexual reproduction on tomato versus asexual reproduction on potato (Lebreton & Andrivon, 1998). However, this cannot explain the current finding because no evidence of sexual reproduction has occurred in *P. infestans* populations on tomato in either China or India (Chowdappa et al., 2014; Yang et al., 2009). The difference more likely reflects the difference in selection pressure imposed by the two crops. In potato, *P. infestans* recurrently moves from and forth between foliages and tubers, posing a strong selection pressure on the pathogen and reducing its genetic variation. On the other hand, no such selection occurs in the tomato production system.

*Phytophthora infestans* is a pathogen with a great potential for international migration (Fry et al., 2015). Successful clonal lineages originating from a regional population can quickly spread globally. For example, Blue_13 first reported in Europe in 2004 has been detected in many parts of the world (Cooke et al., 2012) including China and India (Chowdappa et al., 2014; Li et al., 2013). Interestingly, no nucleotide haplotypes of the eEF‐1α gene were shared between the *P. infestans* populations from China and India, suggesting cross‐border movement of the pathogen in our study may not be as frequent as reported previously (Fry et al., 1993).

India is bordered by the Himalaya Plateau with Tibet of China, and the majority of lands in Tibet areas are not conducive for agricultural production. Furthermore, cross‐border trade of potato and tomato products between the two countries is also very limited. China exports potato and tomato mostly to Russia, Japan, and South Korea (Wang & Zhang, 2004) and potato imports mainly from the USA and Europe (Huang, 2004). On the other hand, the biggest potato export from India goes to Nepal, Sri Lanka, Pakistan, Mauritius, and Bangladesh (Kumarasamy & Sekar, 2014) and Indian potato imports mainly from Germany and France (https://www.infodriveindia.com/). The unique landscape structure coupled with reduced anthropogenic activities related to potato and tomato materials largely disconnect the *P. infestans* populations between the two countries, contributing to the observed population genetic differentiation. Blue‐13 lineage is consisted of many genotypes. Different Blue‐13 genotypes from Europe by different migration events could also generate the observed spatial pattern of haplotype distribution (Chowdappa et al., 2014; Li et al., 2013).

Our results have implications to the management of late blight in potato and tomato. Even though no evidence of direct gene flow was shown to have occurred between the *P. infestans* populations in the two biggest potato production countries in the study reported here, necessary precautions should be taken to prevent the indirect movement of the pathogen through third‐party countries by implementing strict quarantine procedures.

## CONFLICT OF INTEREST

None declared.

## Data Availability

Associated data have been deposited in GenBank: Accession Numbers MN422761–MN422925.

## References

[eva12870-bib-0001] Baldauf, S. L. , & Doolittle, W. F. (1997). Origin and evolution of the slime molds (Mycetozoa). Proceedings of the National Academy of Sciences of the United States of America, 94, 12007–12012. 10.2307/43008 9342353 PMC23686

[eva12870-bib-0002] Balloux, F. , Lugon‐Moulin, N. , & Hausser, J. (2000). Estimating gene flow across hybrid zones: How reliable are microsatellites? Acta Theriologica, 45, 93–101. 10.4098/AT.arch.00-65

[eva12870-bib-0003] Benedick, S. , White, T. A. , Searle, J. B. , Hamer, K. C. , Mustaffa, N. , Khen, C. V. , … Hill, J. K. (2007). Impacts of habitat fragmentation on genetic diversity in a tropical forest butterfly on Borneo. Journal of Tropical Ecology, 23, 623–634. 10.1017/S0266467407004543

[eva12870-bib-0004] Booth Jones, K. A. , Nicoll, M. A. C. , Raisin, C. , Dawson, D. A. , Hipperson, H. , Horsburgh, G. J. , … Norris, K. (2017). Widespread gene flow between oceans in a pelagic seabird species complex. Molecular Ecology, 26, 5716–5728. 10.1111/mec.14330 28833786

[eva12870-bib-0005] Caballero, A. , Quesada, H. , & Rolan‐Alvarez, E. (2008). Impact of amplified fragment length polymorphism size homoplasy on the estimation of population genetic diversity and the detection of selective loci. Genetics, 179, 539–554. 10.1534/genetics.107.083246 18493070 PMC2390631

[eva12870-bib-0006] Cárdenas, M. , Grajales, A. , Sierra, R. , Rojas, A. , González‐Almario, A. , Vargas, A. , … Restrepo, S. (2011). Genetic diversity of *Phytophthora infestans* in the Northern Andean region. BMC Genetics, 12, 23 10.1186/1471-2156-12-23 21303555 PMC3046917

[eva12870-bib-0007] Chang, J. S. , Seok, H. , Kwon, T. K. , Min, D. S. , Ahn, B. H. , Lee, Y. H. , … Suh, P. G. (2002). Interaction of elongation factor‐1alpha and pleckstrin homology domain of phospholipase C‐gamma 1 with activating its activity. Journal of Biological Chemistry, 277, 19697–19702. 10.1074/jbc.M111206200 11886851

[eva12870-bib-0008] Chen, F. P. , Zhou, Q. , Qin, C. F. , Li, Y. , & Zhan, J. (2018). Low evolutionary risk of iprovalicarb resistance in *Phytophthora infestans* . Pesticide Biochemistry and Physiology, 152, 76–83. 10.1016/j.pestbp.2018.09.003 30497714

[eva12870-bib-0009] Chen, Y. , & Halterman, D. A. (2011). Phenotypic characterization of potato late blight resistance mediated by the broad‐spectrum resistance gene *RB* . Phytopathology, 101, 263–270. 10.1094/PHYTO-04-10-0119 20923366

[eva12870-bib-0010] Chowdappa, P. , Nirmal Kumar, B. J. , Madhura, S. , Mohan Kumar, S. P. , Myers, K. L. , Fry, W. E. , & Cooke, D. E. L. (2014). Severe outbreaks of late blight on potato and tomato in South India caused by recent changes in the *Phytophthora infestans* population. Plant Pathology, 64, 191–199. 10.1111/ppa.12228

[eva12870-bib-0011] Cooke, D. E. L. , Cano, L. M. , Raffaele, S. , Bain, R. A. , Cooke, L. R. , Etherington, G. J. , … Kamoun, S. (2012). Genome analyses of an aggressive and invasive lineage of the Irish potato famine pathogen. PLoS Path, 8, e1002940 10.1371/journal.ppat.1002940 PMC3464212 23055926

[eva12870-bib-0012] Cornuet, J. M. , Piry, S. , Luikart, G. , Estoup, A. , & Solignac, M. (1999). New methods employing multilocus genotypes to select or exclude populations as origins of individuals. Genetics, 153, 1989–2000. 10.1017/S0016672399004188 10581301 PMC1460843

[eva12870-bib-0013] Dey, T. , Saville, A. , Myers, K. , Tewari, S. , Cooke, D. E. L. , Tripathy, S. , … Guha Roy, S. (2018). Large sub‐clonal variation in *Phytophthora infestans* from recent severe late blight epidemics in India. Scientific Reports, 8, 4429 10.1038/s41598-018-22192-1 29535313 PMC5849725

[eva12870-bib-0014] Dong, S. , Liu, Y. , Yu, C. , Zhang, Z. , Chen, M. , & Wang, C. (2016). Investigating pollen and gene flow of WYMV‐resistant transgenic wheat N12–1 using a dwarf male‐sterile line as the pollen receptor. PLoS ONE, 11, e0151373 10.1371/journal.pone.0151373 26975052 PMC4790897

[eva12870-bib-0015] Edelaar, P. , & Bolnick, D. I. (2012). Non‐random gene flow: An underappreciated force in evolution and ecology. Trends in Ecology and Evolution, 27, 659–665. 10.1016/j.tree.2012.07.009 22884295

[eva12870-bib-0016] Fernández‐Pavía, S. P. , Grünwald, N. J. , Díaz‐Valasis, M. , Cadena‐Hinojosa, M. , & Fry, W. E. (2004). Soilborne oospores of *Phytophthora infestans* in central Mexico survive winter fallow and infect potato plants in the field. Plant Disease, 88, 29–33. 10.1094/PDIS.2004.88.1.29 30812452

[eva12870-bib-0017] Flier, W. G. , Grunwald, N. J. , Kroon, L. P. , Sturbaum, A. K. , van den Bosch, T. B. , Garay‐Serrano, E. , … Turkensteen, L. J. (2003). The population structure of *Phytophthora infestans* from the Toluca valley of central Mexico suggests genetic differentiation between populations from cultivated potato and wild *Solanum* spp. Phytopathology, 93, 382–390. 10.1094/phyto.2003.93.4.382 18944351

[eva12870-bib-0018] Fry, W. E. , Birch, P. R. J. , Judelson, H. S. , Grünwald, N. J. , Danies, G. , Everts, K. L. , … Smart, C. D. (2015). Five reasons to consider *Phytophthora infestans* a reemerging pathogen. Phytopathology, 105, 966–981. 10.1094/PHYTO-01-15-0005-FI 25760519

[eva12870-bib-0019] Fry, W. , Goodwin, S. B. , Dyer, A. T. , Matuszak, J. M. , Drenth, A. , Tooley, P. W. , … Sandlan, K. P. (1993). Historical and recent migrations of *Phytophthora infestans* : Chronology, pathways, and implications. Glia, 1993(45), 155–169. 10.1094/PD-77-0653

[eva12870-bib-0020] Garant, D. , Forde, S. E. , & Hendry, A. P. (2007). The multifarious effects of dispersal and gene flow on contemporary adaptation. Functional Ecology, 21, 434–443. 10.1111/j.1365-2435.2006.01228.x

[eva12870-bib-0021] Goodwin, S. B. , Sujkowski, L. S. , Dyer, A. T. , Fry, B. A. , & Fry, W. E. (1995). Direct detection of gene flow and probable sexual reproduction of *Phytophthora infestans* in northern North America. Phytopathology, 85, 473–479. 10.1094/Phyto-85-473

[eva12870-bib-0022] Haas, B. J. , Kamoun, S. , Zody, M. C. , Jiang, R. H. Y. , Handsaker, R. E. , Cano, L. M. , … Nusbaum, C. (2009). Genome sequence and analysis of the Irish potato famine pathogen *Phytophthora infestans* . Nature, 461, 393–398. 10.1038/nature08358 19741609

[eva12870-bib-0023] Hall, T. (1999). BioEdit: A user‐friendly biological sequence alignment program for Windows 95/98/NT. Nucleic Acids Symposium Series, 41, 95–98.

[eva12870-bib-0024] Hovemann, B. , Richter, S. , Walldorf, U. , & Cziepluch, C. (1988). Two genes encode related cytoplasmic elongation factors 1 alpha (EF‐1 alpha) in *Drosophila melanogaster* with continuous and stage specific expression. Nucleic Acids Research, 16, 3175–3194. 10.1093/nar/16.8.3175 3131735 PMC336487

[eva12870-bib-0025] Hu, Y. F. , Gao, G. F. , & Zhu, B. L. (2017). The antibiotic resistome: Gene flow in environments, animals and human beings. Frontiers of Medicine, 11, 161–168. 10.1007/s11684-017-0531-x 28500429

[eva12870-bib-0026] Huang, S. (2004). Global trade patterns in fruits and vegetables. USDA International Agriculture and Trade Outlook No. (WRS-0406). pp 88, 10.2139/ssrn.753525

[eva12870-bib-0027] Jordan, I. K. , Rogozin, I. B. , Wolf, Y. I. , & Koonin, E. V. (2002). Essential genes are more evolutionarily conserved than are nonessential genes in bacteria. Genome Research, 12, 962–968. 10.1101/gr.87702 12045149 PMC1383730

[eva12870-bib-0028] Judelson, H. S. , Ah‐Fong, A. M. , Aux, G. , Avrova, A. O. , Bruce, C. , Cakir, C. , … Windass, J. (2008). Gene expression profiling during asexual development of the late blight pathogen *Phytophthora infestans* reveals a highly dynamic transcriptome. Molecular Plant‐Microbe Interactions, 21, 433–447. 10.1094/mpmi-21-4-0433 18321189

[eva12870-bib-0029] Kato, M. V. , Sato, H. , Nagayoshi, M. , & Ikawa, Y. (1997). Upregulation of the elongation factor‐1alpha gene by p53 in association with death of an erythroleukemic cell line. Blood, 90, 1373–1378. 10.1016/j.brainres.2010.10.097 9269753

[eva12870-bib-0030] Knapova, G. , & Gisi, U. (2002). Phenotypic and genotypic structure of *Phytophthora infestans* populations on potato and tomato in France and Switzerland. Plant Pathology, 51, 641–653. 10.1046/j.1365-3059.2002.00750.x

[eva12870-bib-0031] Korman, A. K. , Mallet, J. , Goodenough, J. L. , Graves, J. B. , Hayes, J. L. , Hendricks, D. E. , … Wall, M. (1993). Population structure in *Heliothis virescens* (Lepidoptera: Noctuidae): An estimate of gene flow. Annals of the Entomological Society of America, 86, 182–188. 10.1093/aesa/86.2.182

[eva12870-bib-0032] Kumar, S. , Stecher, G. , & Tamura, K. (2016). MEGA7: Molecular evolutionary genetics analysis version 7.0 for bigger datasets. Molecular Biology and Evolution, 33, 1870–1874. 10.1093/molbev/msw054 27004904 PMC8210823

[eva12870-bib-0033] Kumarasamy, N. , & Sekar, C. (2014). Export of fresh and frozen potatoes from India – An economic analysis. International Journal of Processing and Post Harvest Technology, 5, 173–178. 10.15740/HAS/IJPPHT/5.2/173-178

[eva12870-bib-0034] Lázaro‐Nogal, A. , Matesanz, S. , García‐Fernández, A. , Traveset, A. , & Valladares, F. (2017). Population size, center–periphery, and seed dispersers' effects on the genetic diversity and population structure of the Mediterranean relict shrub *Cneorum tricoccon* . Ecology and Evolution, 7, 7231–7342. 10.1002/ece3.2940 28944013 PMC5606867

[eva12870-bib-0035] Lebreton, L. , & Andrivon, D. (1998). French isolates of *Phytophthora infestans* from potato and tomato differ in phenotype and genotype. European Journal of Plant Pathology, 104, 583–594. 10.1023/A:1008662518345

[eva12870-bib-0036] Lees, A. K. , Wattier, R. , Shaw, D. S. , Sullivan, L. , Williams, N. A. , & Cooke, D. E. L. (2006). Novel microsatellite markers for the analysis of *Phytophthora infestans* populations. Plant Pathology, 55, 311–319. 10.1111/j.1365-3059.2006.01359.x

[eva12870-bib-0037] Lexer, C. , Marthaler, F. , Humbert, S. , Barbará, T. , Harpe, M. D. L. , Bossolini, E. , … Versieux, L. M. (2016). Gene flow and diversification in a species complex of *Alcantarea* inselberg bromeliads. Botanical Journal of the Linnean Society, 181, 505–520. 10.1111/boj.12372

[eva12870-bib-0038] Li, Y. , van der Lee, T. , Zhu, J. H. , Jin, G. H. , Lan, C. Z. , Zhu, S. X. , … Jacobsen, E. (2013). Population structure of *Phytophthora infestans* in China – Geographic clusters and presence of the EU genotype Blue_13. Plant Pathology, 62, 932–942. 10.1111/j.1365-3059.2012.02687.x

[eva12870-bib-0039] Librado, P. J. R. , & Rozas, J. (2009). DnaSP v5: A software for comprehensive analysis of DNA polymorphism data. Bioinformatics, 25, 1451–1452. 10.1093/bioinformatics/btp187 19346325

[eva12870-bib-0040] McDonald, B. A. , & Linde, C. (2002). The population genetics of plant pathogens and breeding strategies for durable resistance. Euphytica, 124, 163–180. 10.1023/A:1015678432355

[eva12870-bib-0041] Meirmans, P. G. , & Hedrick, P. W. (2011). Assessing population structure: *F* _ST_ and related measures. Molecular Ecology Resources, 11, 5–18. 10.1111/j.1755-0998.2010.02927.x 21429096

[eva12870-bib-0042] Miller, M. E. , Zhang, Y. , Omidvar, V. , Sperschneider, J. , Schwessinger, B. , Raley, C. , … Figueroa, M. (2018). *De novo* assembly and phasing of dikaryotic genomes from two isolates of *Puccinia coronata* f. sp. *avenae*, the causal agent of oat crown rust. American Society for Microbiology, 9, e01650‐01617 10.1128/mBio.01650-17 PMC5821079 29463655

[eva12870-bib-0043] Moldave, K. (1985). Eukaryotic protein synthesis. Annual Review of Biochemistry, 54, 1109–1149. 10.1146/annurev.bi.54.070185.005333 3896117

[eva12870-bib-0044] Montarry, J. , Andrivon, D. , Glais, I. , Corbiere, R. , Mialdea, G. , & Delmotte, F. (2010). Microsatellite markers reveal two admixed genetic groups and an ongoing displacement within the French population of the invasive plant pathogen *Phytophthora infestans* . Molecular Ecology, 19, 1965–1977. 10.1111/j.1365-294X.2010.04619.x 20345671

[eva12870-bib-0045] Nirmal Kumar, B. J. , Chowdappa, P. , & Krishna, V. (2016). Multilocus phylogenetic analysis of *Phytophthora infestans* isolates infecting potato and tomato in India. Indian Phytopathology, 69, 38–46.

[eva12870-bib-0046] O'Donald, P. (1972). Evolution and the genetics of populations. Volume 2. The theory of gene frequencies. By Sewall Wright. Journal of Biosocial Science, 4, 253–256. 10.1017/s0021932000008543

[eva12870-bib-0047] Panyamang, A. , Duangphakdee, O. , & Rattanawannee, A. (2018). Genetic structure of teak beehole borer, *Xyleutes ceramicus* (Lepidoptera: Cossidae), in northern Thailand. Agriculture and Natural Resources, 52, 66–74. 10.1016/j.anres.2018.05.008

[eva12870-bib-0048] Paun, O. , Schönswetter, P. , Winkler, M. , Tribsch, A. , & Intrabiodiv, C. (2008). Historical divergence vs. contemporary gene flow: evolutionary history of the calcicole *Ranunculus alpestris* group (Ranunculaceae) in the European Alps and the Carpathians. Molecular Ecology, 17, 4263–4275. 10.1111/j.1365-294X.2008.03908.x 19378404 PMC2988486

[eva12870-bib-0049] Piedra‐Quintero, Z. L. , Apodaca‐Medina, A. I. , Beltran‐Lopez, E. , Leon‐Sicairos, C. R. , & Lopez‐Moreno, H. S. (2015). Immunoproteomic Identification of p29 Antigen as the Elongation Factor‐1a of *Leishmania mexicana* . Vector‐Borne and Zoonotic Diseases, 15, 449–452. 10.1089/vbz.2014.1712 26186518

[eva12870-bib-0050] Ralph, P. L. , & Coop, G. (2015). Convergent evolution during local adaptation to patchy landscapes. PLOS Genetics, 11, e1005630 10.1371/journal.pgen.1005630 26571125 PMC4646681

[eva12870-bib-0051] Rannala, B. , & Mountain, J. L. (1997). Detecting immigration by using multilocus genotypes. Proceedings of the National Academy of Sciences of the United States of America, 94, 9197–9201. 10.1073/pnas.94.17.9197 9256459 PMC23111

[eva12870-bib-0052] Riis, B. , Rattan, S. I. S. , Clark, B. F. C. , & Merrick, W. C. (1990). Eukaryotic protein elongation factors. Trends in Biochemical Sciences, 15, 420–424. 10.1016/0968-0004(90)90279-K 2278101

[eva12870-bib-0053] Ropars, J. , Maufrais, C. , Diogo, D. , Marcet‐Houben, M. , Perin, A. , Sertour, N. , … d'Enfert, C. (2018). Gene flow contributes to diversification of the major fungal pathogen *Candida albicans* . Nature Communications, 9, 2253 10.1038/s41467-018-04787-4 PMC5993739 29884848

[eva12870-bib-0054] Runno‐Paurson, E. , Hannukkala, A. O. , Kotkas, K. , Koppel, M. , Williams, I. H. , & Mänd, M. (2013). Impact of phytosanitary quality of seed potato and temporal epidemic progress on the phenotypic diversity of *Phytophthora infestans* populations. American Journal of Potato Research, 90, 245–254. 10.1007/s12230-013-9299-y

[eva12870-bib-0055] Saitou, N. , & Nei, M. (1987). The neighbor‐joining method: A new method for reconstructing phylogenetic trees. Molecular Biology and Evolution, 4, 406–425. 10.1093/oxfordjournals.molbev.a040454 3447015

[eva12870-bib-0056] Semizer‐Cuming, D. , Kjaer, E. D. , & Finkeldey, R. (2017). Gene flow of common ash (*Fraxinus excelsior* L.) in a fragmented landscape. PLoS ONE, 12, e0186757 10.1371/journal.pone.0186757 29053740 PMC5650178

[eva12870-bib-0057] Singh, P. , & Singh, B. N. (2008). Population genetics of *Drosophila ananassae* . Genetics Research, 90, 409 10.1017/S0016672308009737 19061531

[eva12870-bib-0058] Slatkin, M. (1987). Gene flow and the geographic structure of natural populations. Science, 236, 787–792. 10.1126/science.3576198 3576198

[eva12870-bib-0059] Stergiopoulos, I. , Cordovez, V. , Okmen, B. , Beenen, H. G. , Kema, G. H. , & de Wit, P. J. (2013). Positive selection and intragenic recombination contribute to high allelic diversity in effector genes of *Mycosphaerella fijiensis*, causal agent of the black leaf streak disease of banana. Molecular Plant Pathology, 15, 447–460. 10.1111/mpp.12104 24245940 PMC6638713

[eva12870-bib-0060] Tian, Y. E. , Yin, J. L. , Sun, J. P. , Ma, H. M. , Ma, Y. F. , Quan, J. L. , & Shan, W. X. (2015). Population structure of the late blight pathogen *Phytophthora infestans* in a potato germplasm nursery in two consecutive years. Phytopathology, 105, 771–777. 10.1094/PHYTO-03-14-0073-R 25738550

[eva12870-bib-0061] Tian, Y. E. , Yin, J. L. , Sun, J. P. , Ma, Y. F. , Ma, Y. F. , Wang, Q. H. , … Shan, W. X. (2015). Population genetic analysis of Phytophthora infestans in northwestern China. Plant Pathology, 65, 17–25. 10.1111/ppa.12392

[eva12870-bib-0062] Toews, D. P. , Mandic, M. , Richards, J. G. , & Irwin, D. E. (2013). Migration, mitochondria, and the yellow‐rumped warbler. Evolution, 68, 241–255. 10.1111/evo.12260 24102562

[eva12870-bib-0063] van't Klooster, J. W. , van den Berg‐Velthuis, G. , van West, P. , & Govers, F. (2000). tef1, a *Phytophthora infestans* gene encoding translation elongation factor 1alpha. Gene, 249, 145–151. 10.1016/S0378-1119(00)00151-7 10831848

[eva12870-bib-0064] Viscidi, R. P. , & Demma, J. C. (2003). Genetic diversity of *Neisseria gonorrhoeae* housekeeping genes. Journal of Clinical Microbiology, 41, 197–204. 10.1128/JCM.41.1.197-204.2003 12517848 PMC149597

[eva12870-bib-0065] Wang, Q. B. , & Zhang, W. (2004). China's potato industry and potential impacts on the global market. American Journal of Potato Research, 81, 101–109. 10.1007/bf02853607

[eva12870-bib-0066] Wangsomboondee, T. , Groves, C. T. , Shoemaker, P. B. , Cubeta, M. A. , & Ristaino, J. B. (2002). *Phytophthora infestans* populations from tomato and potato in North Carolina differ in genetic diversity and structure. Phytopathology, 92, 1189–1195. 10.1094/PHYTO.2002.92.11.1189 18944244

[eva12870-bib-0067] Watt, W. B. (1972). Intragenic recombination as a source of population genetic variability. The American Naturalist, 106, 737–753. 10.1086/282809

[eva12870-bib-0068] Wright, S. (1990). Evolution in Mendelian populations. Bulletin of Mathematical Biology, 52, 241–259. 10.1007/BF02459575 2185860

[eva12870-bib-0069] Yang, L. N. , Ouyang, H. B. , Fang, Z. G. , Zhu, W. , Wu, E. J. , Luo, G. H. , … Zhan, J. (2018). Evidence for intragenic recombination and selective sweep in an effector gene of *Phytophthora infestans* . Evolutionary Applications, 11, 1342–1353. 10.1111/eva.12629 30151044 PMC6099815

[eva12870-bib-0070] Yang, L. N. , Zhu, W. , Wu, E. J. , Yang, C. , Thrall, P. H. , Burdon, J. J. , … Zhan, J. (2016). Trade‐offs and evolution of thermal adaptation in the Irish potato famine pathogen *Phytophthora infestans* . Molecular Ecology, 25, 4047–4058. 10.1111/mec.13727 27288627

[eva12870-bib-0071] Yang, Z. H. , Zhu, J. H. , Shen, J. W. , Yao, G. S. , Rui, L. , Forbes, G. A. , Fry, W. E. (2009). Microsatellite genotypic analysis of phytophthora infestans in china. Acta Horticulturae, 834), 187–192. 10.17660/ActaHortic.2009.834.20

[eva12870-bib-0072] Zhan, J. , Thrall, P. H. , & Burdon, J. J. (2014). Achieving sustainable plant disease management through evolutionary principles. Trends in Plant Science, 19, 570–575. 10.1016/j.tplants.2014.04.010 24853471

[eva12870-bib-0073] Zhan, J. , Thrall, P. H. , Papaix, J. , Xie, L. , & Burdon, J. J. (2015). Playing on a pathogen's weakness: using evolution to guide sustainable plant disease control strategies. Annual Review of Phytopathology, 53, 19–43. 10.1146/annurev-phyto-080614-120040 25938275

[eva12870-bib-0074] Zhu, W. , Shen, L.‐L. , Fang, Z.‐G. , Yang, L.‐N. , Zhang, J.‐F. , Sun, D.‐L. , & Zhan, J. (2016). Increased frequency of self‐fertile isolates in *Phytophthora infestans* may attribute to their higher fitness relative to the A1 isolates. Scientific Reports, 6, 29428 10.1038/srep29428 27384813 PMC4935937

[eva12870-bib-0075] Zhu, W. , Yang, L. N. , Wu, E. J. , Qin, C. F. , Shang, L. P. , Wang, Z. H. , & Zhan, J. (2015). Limited sexual reproduction and quick turnover in the population genetic structure of *Phytophthora infestans* in Fujian, China. Scientific Reports, 5, 10094 10.1038/srep10094 25970264 PMC4429539

