# Supplementary material for: Lack of gene flow between Phytophthora infestans populations of two neighboring countries with the largest potato production
Source: Evol Appl. 2019 Sep 28;13(2):318–29. doi: 10.1111/eva.12870 (PMC6976962; doi:10.1111/eva.12870)
Supplement: Supplementary file 1 [file EVA-13-318-s001.docx]

Table S1 Sequence accession number and original code of the Indian isolates stored in Genbank

| Acc. number | Code | Country | Host |
| --- | --- | --- | --- |
| KR046590 | PIP7 | India | potato |
| KR046591 | PIP10 | India | potato |
| KR046592 | PIP19 | India | potato |
| KR046593 | PIP24 | India | potato |
| KR046594 | PIP28 | India | potato |
| KR046595 | PIP30 | India | potato |
| KR046596 | PIP32 | India | potato |
| KR046597 | PIP33 | India | potato |
| KR046598 | PIP40 | India | potato |
| KR046599 | PIP44 | India | potato |
| KR046600 | PIP48 | India | potato |
| KR046601 | PIP49 | India | potato |
| KR046602 | PIP52 | India | potato |
| KR046603 | PIP53 | India | potato |
| KR046604 | PIP56 | India | potato |
| KR046605 | PIP58 | India | potato |
| KR046606 | PIP59 | India | potato |
| KR046607 | PIP62 | India | potato |
| KR046608 | PIP64 | India | potato |
| KR046609 | PIP76 | India | potato |
| KR046610 | PIP77 | India | potato |
| KR046611 | PIP78 | India | potato |
| KR046612 | PIP79 | India | potato |
| KR046613 | PIP80 | India | potato |
| KR046614 | PIP81 | India | potato |
| KR046615 | PIP82 | India | potato |
| KR046616 | PIP83 | India | potato |
| KR046617 | PIP84 | India | potato |
| KR046618 | PIP85 | India | potato |
| KR046619 | PIP86 | India | potato |
| KR046620 | PIP87 | India | potato |
| KR046621 | PIP88 | India | potato |
| KR046622 | PIP89 | India | potato |
| KR046623 | PIP90 | India | potato |
| KR046624 | PIP91 | India | potato |
| KR046625 | PIP92 | India | potato |
| KR046626 | PIP93 | India | potato |
| KR046627 | PIP94 | India | potato |
| KR046628 | PIP95 | India | potato |
| KR046629 | PIP96 | India | potato |
| KR046630 | PIP97 | India | potato |
| KR046631 | PIP98 | India | potato |
| KR046632 | PIP99 | India | potato |
| KR046633 | PIP100 | India | potato |
| KR046634 | PIP101 | India | potato |
| KR046635 | PIP102 | India | potato |
| KR046636 | PIP103 | India | potato |
| KR046637 | PIP104 | India | potato |
| KR046638 | PIT17 | India | tomato |
| KR046639 | PIT19 | India | tomato |
| KR046640 | PIT21 | India | tomato |
| KR046641 | PIT22 | India | tomato |
| KR046642 | PIT25 | India | tomato |
| KR046643 | PIT26 | India | tomato |
| KR046644 | PIT27 | India | tomato |
| KR046645 | PIT28 | India | tomato |
| KR046646 | PIT29 | India | tomato |
| KR046647 | PIT30 | India | tomato |
| KR046648 | PIT31 | India | tomato |
| KR046649 | PIT36 | India | tomato |
| KR046650 | PIT38 | India | tomato |
| KR046651 | PIT39 | India | tomato |
| KR046652 | PIT40 | India | tomato |
| KR046653 | PIT41 | India | tomato |
| KR046654 | PIT42 | India | tomato |
| KR046655 | PIT43 | India | tomato |
| KR046656 | PIT44 | India | tomato |
| KR046657 | PIT46 | India | tomato |
| KR046658 | PIT47 | India | tomato |
| KR046659 | PIT48 | India | tomato |
| KR046660 | PIT49 | India | tomato |
| KR046661 | PIT50 | India | tomato |
| KR046662 | PIT51 | India | tomato |
| KR046663 | PIT52 | India | tomato |
| KR046664 | PIT53 | India | tomato |
| KR046666 | PIT55 | India | tomato |
| KR046667 | PIT56 | India | tomato |
| KR046668 | PIT59 | India | tomato |
| KR046669 | PIT70 | India | tomato |
| KR046670 | PIT84 | India | tomato |
